# Genetic association of hypoxia inducible factor 1-alpha (*HIF1A*) Pro582Ser polymorphism with risk of diabetes and diabetic complications

**DOI:** 10.18632/aging.103213

**Published:** 2020-07-13

**Authors:** Huan Ren, Jian-Quan Luo, Yong-Chao Gao, Man-Yun Chen, Xiao-Ping Chen, Hong-Hao Zhou, Ying Jiang, Wei Zhang

**Affiliations:** 1 Department of Clinical Pharmacology, Xiangya Hospital, Central South University, Changsha, P.R. China; 2 Institute of Clinical Pharmacology, Central South University, Hunan Key Laboratory of Pharmacogenetics, Changsha, P.R. China; 3 Engineering Research Center of Applied Technology of Pharmacogenomics, Ministry of Education, Changsha, P.R. China; 4 National Clinical Research Center for Geriatric Disorders, Changsha, P.R. China; 5 Department of Pharmacy, The Second Xiangya Hospital, Central South University, Changsha, P.R. China; 6 Department of Cardiothoracic Surgery, Xiangya Hospital, Central South University, Changsha, P.R. China

**Keywords:** diabetes, diabetic complications, HIF1A, polymorphism, Pro582Ser

## Abstract

Diabetes is an age-related chronic disease associated with a number of complications, emerging as one of the major causes of morbidity and mortality worldwide. Several studies indicated that hypoxia-inducible factor 1-alpha *(HIF1A*) genetic polymorphisms may be associated with diabetes and diabetic complications. However, this association remains ambiguous. Thus, we performed a meta-analysis to provide more precise conclusion on this issue. Odds ratios (OR) with corresponding 95% confidence intervals (CI) were applied to assess the strength of the relationships. There was a protective association between *HIF1A* Pro582Ser polymorphism and diabetes under the heterozygous genetic model (OR = 0.70, 95% CI = 0.55-0.91; *P* = 0.007). Similar associations were observed in diabetic complications risk under the allelic (OR = 0.69, 95% CI = 0.57-0.83; *P* < 0.001), homozygous (OR = 0.51, 95% CI = 0.30-0.87; *P* = 0.014), recessive (OR = 0.73, 95% CI = 0.59-0.90; *P* = 0.004) and dominant (OR = 0.40, 95% CI = 0.25-0.65; *P* < 0.001) genetic models. No effects of the *HIF1A* Ala588Thr polymorphism were found in risk of diabetes and diabetic complications. Taken together, these findings revealed the protective effect of *HIF1A* Pro582Ser polymorphism against diabetes and diabetic complications.

## INTRODUCTION

Diabetes is an age-related chronic disease and has already become one of the major causes of mortality and morbidity worldwide [1]. The progression of diabetes and the occurrence of diabetic complications are largely influenced by early glycemic control [2]. The poor blood glucose control is the major risk factor for diabetic complications, including nephropathy, retinopathy, and neuropathy. After years of careful studies, there is now clear evidence for the genetic susceptibility to diabetes and its complications [3–5]. Genetic studies may offer an opportunity to explore the pathobiology of these diseases.

Hypoxia-inducible factor-1(HIF-1), a master regulator of oxygen homeostasis, allows the adaptive responses to the hypoxic environment [6]. HIF-1 acts as a heterodimer consisting of the HIF-1α and HIF-1β subunit [7]. In normoxia, the regulation of HIF-1 activity is critically dependent on the degradation of the HIF-1a subunit. The molecular basis of HIF-1α degradation is oxygen-dependent hydroxylation of two proline residues (Pro402 or Pro564, or both) that binds to the von Hippel-Lindau tumor-suppressor protein (VHL). VHL recruits E3 ubiquitin-protein ligase complex and targets HIF-1α for proteasomal degradation [8, 9]. Under condition of hypoxia, HIF-1α is stabilized against degradation, which upregulates a series of genes involved in lots of biologic processes such as glycolysis, angiogenesis, erythropoiesis, and age-related diseases [10–14].

Clinical and experimental studies indicate that hyperglycemia suggests a state of pseudohypoxia and activates HIF-1α activity for adaptation of hypoxia [15, 16]. In addition, hyperglycemia may impair the stabilization and transactivation of HIF-1α [17–19]. It has been postulated that the function of HIF-1a is repressed by hyperglycemia leading to the loss of cellular adaptation to hypoxia in diabetes, which suggests a mechanism in the pathophysiology of diabetes and diabetic complications [20–23].

The gene *HIF1A* (for HIF-1α) carries two common nonsynonymous single nucleotide polymorphisms (SNP) in exon 12, Pro582Ser (rs11549465) and Ala588Thr (rs11549467), which both exhibit higher transcriptional activity of *HIF1A* [24, 25]. Previous studies indicated that *HIF1A* Pro582Ser and Ala588Thr may be associated with diabetes and diabetic complications. Yamada et al. [26] firstly reported the *HIF1A* Pro582Ser polymorphism exerted a protective effect in the occurrence of diabetes, but no correlation with diabetic complications in a Japanese population. While Ekberg et al. [27] identified the protective effect of *HIF1A* Pro582Ser on the development of severe diabetic retinopathy with risk reduction of 95%. Several other studies also focused on the associations of *HIF1A* Pro582Ser and Ala588Thr polymorphisms with diabetes and diabetic complications, including type 1 and type 2 diabetes, diabetic nephropathy, diabetic retinopathy and diabetic foot ulcers [28–33]. Nevertheless, the results of these studies are conflicting. To obtain accurate conclusion, we conducted a comprehensive meta-analysis based on the controversial results from various independent case-control studies.

## RESULTS

### Description of eligible studies

**Figure 1:**
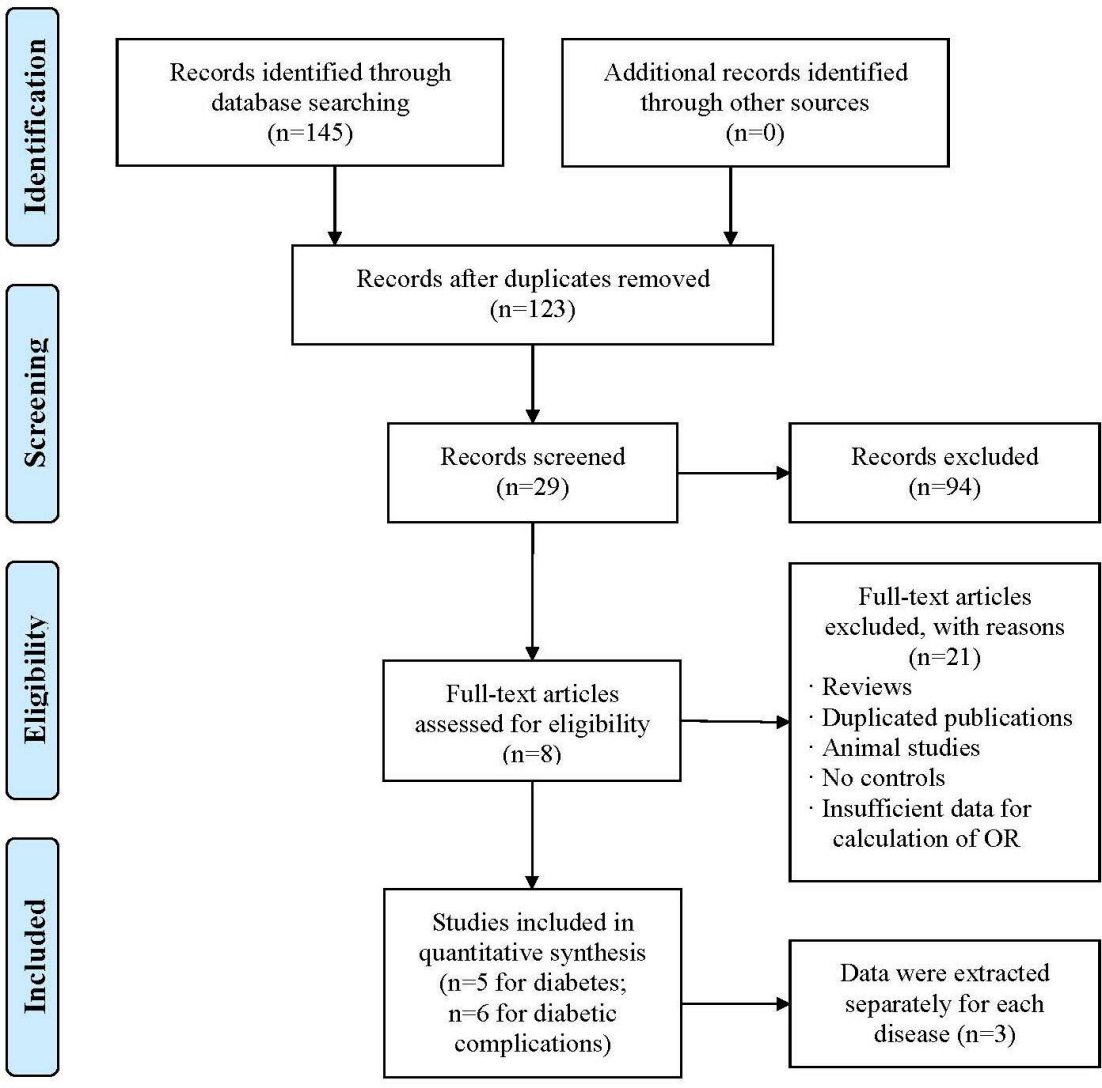
**Flow diagram of the search strategy and study selection. The terms “n” in the boxes represent the number of corresponding studies.**

The current meta-analysis was conducted according to the guidelines of the “Preferred Reporting Items for Systematic reviews and Meta-Analyses’’ (PRISMA) statement. As depicted in the flow diagram (Figure 1), the initial literature screening yielded 145 articles, and a total of 22 articles were excluded due to duplicate publication. Then, 94 articles were removed from screening according to titles and/or abstracts. Finally, based on the study inclusion criteria, 8 articles [26–33] involving 11 eligible studies for the association of *HIF1A* Pro582Ser and Ala588Thr polymorphisms with diabetes and diabetic complications were included in our meta-analysis. All the included articles were all conducted with case-control design and the sample sizes varied from 145 to 1165. A total of 5 and 6 eligible studies were identified for diabetes and diabetic complications, respectively. The general characteristics of the studies included in the meta-analysis were presented in Table 1.

**Table 1:** Characteristics of the included studies of association of *HIF1A* Pro582Ser (rs11549465) and Ala588Thr (rs11549467) genetic polymorphisms with diabetes and diabetic complications.

**First author**	**Year**	**Country**	**Male/Female**		**Age(years)**		**Sample size^a^**	**Case genotypes or alleles^b^**					**Control genotypes or alleles^b^**					**Genotyping method**	**HWE-*P*^c^**
			**case**	**control**	**case**	**control**		**11**	**12**	**22**	**1**	**2**	**11**	**12**	**22**	**1**	**2**		
*HIF1A* Pro582Ser (rs11549465) and Diabetes risk																			
Yamada	2005	Japan	245/195	231/342	60.5±11.4	67.3 ±6.5	440/572	404	36	0	844	36	494	72	6	1060	84	Sequencing	0.073
Nagy	2009	Hungary	246/290	115/239	55.6± 7.6	25.1 ± 8.5	536/354	446	87	3	979	93	269	76	9	614	94	PCR-RFLP	0.203
Pichu	2015	India	NA	NA	53.8±11.4	41.9±11.5	79/66	21	18	40	60	98	24	13	29	61	71	PCR-RFLP	9.33e^-07^
*HIF1A* Pro582Ser (rs11549465) and Diabetic complications risk																			
GU	2013	America	311/260	240/354	44.0±6.0	40.0±8.0	594/571	439	148	7	1026	162	453	114	4	1020	122	TaqMan	0.270
Bi	2015	China	72/68	62/42	54.8±14.8	54.6±14.9	140/104	130	10	0	270	10	88	16	0	192	16	PCR-RFLP	0.395
Pichu	2015	India	NA	NA	57.4±9.9	53.8±11.4	79/79	21	18	40	60	98	19	40	20	78	80	PCR-RFLP	0.909
Ekberg	2019	Sweden	318/237	80/68	48.4±0.9	44.9 ± 1.3	148/555	118	21	9	257	39	473	66	16	1012	98	TaqMan	7.45e^-10^
*HIF1A* Ala588Thr(rs11549467) and Diabetes risk																			
Yamada	2005	Japan	245/195	231/342	60.5±11.4	67.3 ±6.5	440/572	400	39	1	839	41	524	46	2	1094	50	Sequencing	0.364
Pichu	2018	India	NA	NA	NA	NA	185/145	48	79	58	175	195	68	24	53	160	130	PCR-RFLP	1.13e^-15^
*HIF1A* Ala588Thr(rs11549467) and Diabetic complications risk																			
Zhao	2016	China	102/98	94/106	54.3±10.8	50.7±11.0	200/200	146	50	4	342	58	118	71	11	307	93	Sequencing	0.940
Pichu	2018	India	NA	NA	NA	NA	199/185	41	82	76	164	234	48	79	58	175	195	PCR-RFLP	0.051

Abbreviations: NA, not available; PCR-RFLP, polymerase chain reaction-restriction fragment length polymorphism.

^a^Sample size means the case/control groups;

^b^For the *HIF1A* Pro582Ser(rs11549465) polymorphism, 1: C, 2: T; 11: CC, 12: CT, 22: TT; For the *HIF1A* Ala588Thr(rs11549467) polymorphism, 1: G, 2: A; 11: GG, 12:GA, 22: AA;

^c^HWE, Hardy-Weinberg equilibrium in control group.

### Quantitative synthesis of the association between *HIF1A* Pro582Ser polymorphism and the risk of diabetes

**Figure 2:**
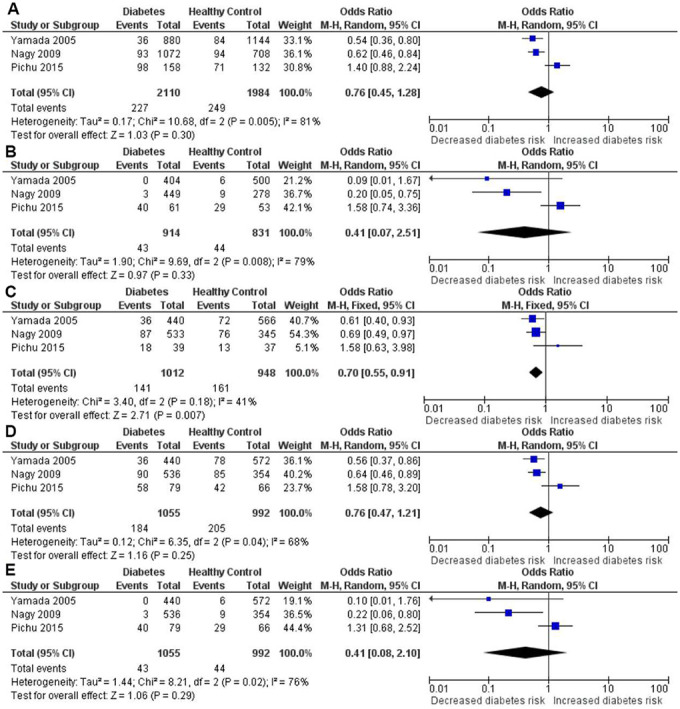
Forest plot of the meta-analysis for the association between the *HIF1A* Pro582Ser genetic polymorphism and diabetes risk under the allelic (**A**), homozygous (**B**), heterozygous (**C**), dominant (**D**) and recessive (**E**) genetic model.

The results of meta-analysis and heterogeneity test between *HIF1A* Pro582Ser polymorphism and the risk of diabetes were summarized in detail in Table 2 and Figure 2. The pooled analysis revealed a significant protective effect of the Pro582Ser polymorphism on the risk of diabetes under the heterozygous genetic model (OR = 0.70, 95% CI = 0.55-0.91; *P* = 0.007). Furthermore, both the Cochran’s Q test and estimate of I^2^ showed no significant between-study heterogeneity under the heterozygous genetic model (*P*_heterogeneity_ = 0.813, I^2^ = 41.2%). In contrast, no significant association was found between *HIF1A* Pro582Ser polymorphism and diabetes under the allelic (OR = 0.76, 95% CI = 0.45-1.28; *P* = 0.301), homozygous (OR = 0.41, 95% CI = 0.07-2.51; *P* = 0.333), recessive (OR = 0.76, 95% CI = 0.47-1.22; *P* = 0.248) and dominant (OR = 0.41, 95% CI = 0.08-2.10; *P* = 0.287) genetic models. However, significant heterogeneity among the constituent studies was found under the allelic (*P*_heterogeneity_ = 0.005, I^2^ = 81.3%), homozygous (*P*_heterogeneity_ = 0.008, I^2^ = 79.4%), recessive (*P*_heterogeneity_ = 0.042, I^2^ = 68.5%) and dominant (*P*_heterogeneity_ = 0.017, I^2^ = 75.6%) genetic models.

**Table 2:** Summary of meta-analysis of association of *HIF1A* Pro582Ser (rs11549465) genetic polymorphism with risk of diabetes and diabetic complications.

**Genetic model**	**Pooled analysis**					**Tests of heterogeneity**	
	**Pooled OR(95%CI)**	**Z-value**	***P*-value**	**N**	**Model**	***P*-value**	**I^2^%**
Diabetes risk							
Allelic genetic model	0.76 (0.45-1.28)	1.03	0.301	3	R	0.005	81.30%
Homozygous genetic model	0.41 (0.07-2.51)	0.97	0.333	3	R	0.008	79.40%
Heterozygous genetic model	0.70 (0.55-0.91)	2.71	**0.007**	3	F	0.183	41.20%
Dominant genetic model	0.76 (0.47-1.22)	1.16	0.248	3	R	0.042	68.50%
Recessive genetic model	0.41 (0.08-2.10)	1.06	0.287	3	R	0.017	75.60%
Diabetic complications risk						
Allelic genetic model	0.69 (0.57-0.83)	3.94	**<0.001**	4	F	0.577	0.00%
Homozygous genetic model	0.51 (0.30-0.87)	2.46	**0.014**	3	F	0.923	0.00%
Heterozygous genetic model	0.85 (0.51-1.41)	0.63	0.532	4	R	0.024	68.20%
Dominant genetic model	0.73 (0.59-0.90)	2.89	**0.004**	4	F	0.358	7.00%
Recessive genetic model	0.40 (0.25-0.65)	3.72	**<0.001**	3	F	0.671	0.00%

Abbreviations: CI: confidence interval; F: fixed-effects model; N: the number of the studies in the meta-analysis; OR: odds ratio; R: random-effects model; Allelic genetic model: T vs. C; Homozygous genetic model: TT vs. CC; Heterozygous genetic model: CT vs. CC; Dominant genetic model: TT + CT vs. CC; Recessive genetic model: TT vs. CT + CC. Significant *P*-value in the pooled analysis are in bold.

**Figure 3:**
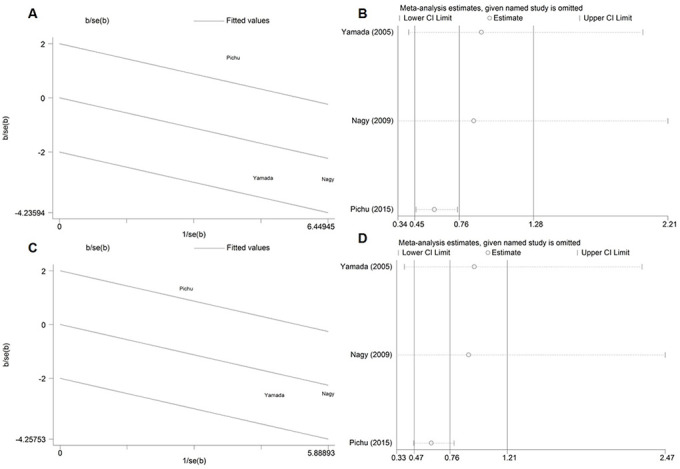
Galbraith plot and sensitivity analysis for the association between the *HIF1A* Pro582Ser genetic polymorphism and diabetes risk under the allelic (**A**, **B**) and dominant (**C**, **D**) genetic model.

Therefore, as shown in the Figure 3, Galbraith plot analysis and sensitivity analysis were performed to detect the possible sources of heterogeneity under the allelic, homozygous, recessive and dominant genetic models. Under the above four genetic models, Galbraith plot analysis revealed that the Pichu et al. study was the outlier, which was consistent with the results of sensitivity analysis. When omitting the study by Pichu et al. in the meta-analysis, no heterogeneity existed under the allelic (*P*_heterogeneity_ = 0.579, I^2^ = 0%), homozygous (*P*_heterogeneity_ = 0.628, I^2^ = 0%), recessive (*P*_heterogeneity_ = 0.649, I^2^ = 0%) and dominant (*P*_heterogeneity_ = 0.619, I^2^ = 0%) genetic models (Table 3). The study by Pichu et al. may be the source of heterogeneity in the meta-analysis for the allelic, homozygous, recessive and dominant genetic models.

**Figure 4:**
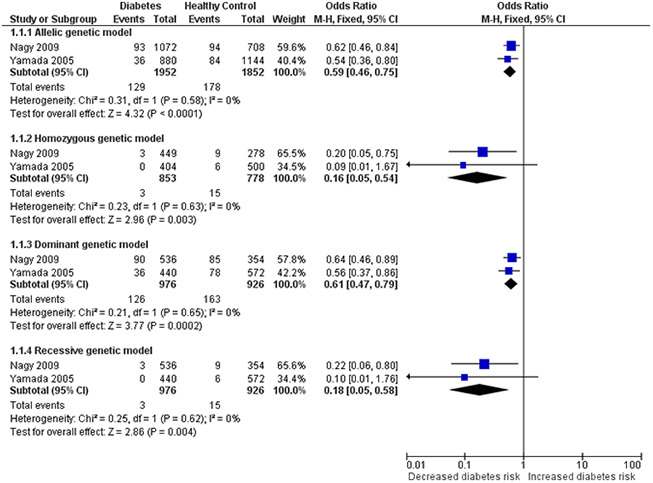
****Forest plot for the meta-analysis of association of the *HIF1A* Pro582Ser genetic polymorphism with risk of diabetes after omitting the outlier under the allelic (**A**), homozygous (**B**), dominant (**C**) and recessive (**D**) genetic model.

In addition, as shown in the Table 3 and Figure 4, the corrected OR also indicated a significant association between *HIF1A* Pro582Ser polymorphism and reduced risk of diabetes under the allelic (OR = 0.59, 95% CI = 0.46-0.75; *P* < 0.001), homozygous (OR = 0.16, 95% CI = 0.05-0.54; *P* = 0.003), recessive (OR = 0.61, 95% CI = 0.47-0.79; *P* < 0.001) and dominant (OR = 0.18, 95% CI = 0.05-0.58; *P* = 0.004) genetic models.

**Table 3:** Summary of meta-analysis of association of the *HIF1A* Pro582Ser (rs11549465) genetic polymorphism with risk of diabetes and diabetic complications after omitting the outlier.

**Genetic model**	**Pooled analysis**						**Tests of heterogeneity**		**Omitted study**
	**Pooled OR(95%CI)**	**Z-value**	***P*-value**	**N**	**Model**		***P*-value**	**I^2^%**	
Diabetes risk									
Allelic genetic model	0.59 (0.46-0.75)	4.32	**<0.001**	2	F		0.579	0.00%	Pichu (2015)
Homozygous genetic model	0.16 (0.05-0.54)	2.96	**0.003**	2	F		0.628	0.00%	Pichu (2015)
Recessive genetic model	0.61 (0.47-0.79)	3.77	**<0.001**	2	F		0.649	0.00%	Pichu (2015)
Dominant genetic model	0.18 (0.05-0.58)	2.86	**0.004**	2	F		0.619	0.00%	Pichu (2015)
Diabetic complications risk									
Heterozygous genetic model	0.72 (0.57-0.91)	2.75	**0.006**		F		0.423	0.00%	Pichu (2015)

Abbreviations: CI: confidence interval; F: fixed-effects model; N: the number of the studies in the meta-analysis; OR: odds ratio; Allelic genetic model: T vs. C; Homozygous genetic model: TT vs. CC; Heterozygous genetic model: CT vs. CC; Dominant genetic model: TT + CT vs. CC; Recessive genetic model: TT vs. CT + CC. Significant *P*-value in the pooled analysis are in bold.

### Quantitative synthesis of the association between *HIF1A* Pro582Ser polymorphism and the risk of diabetic complications

**Figure 5:**
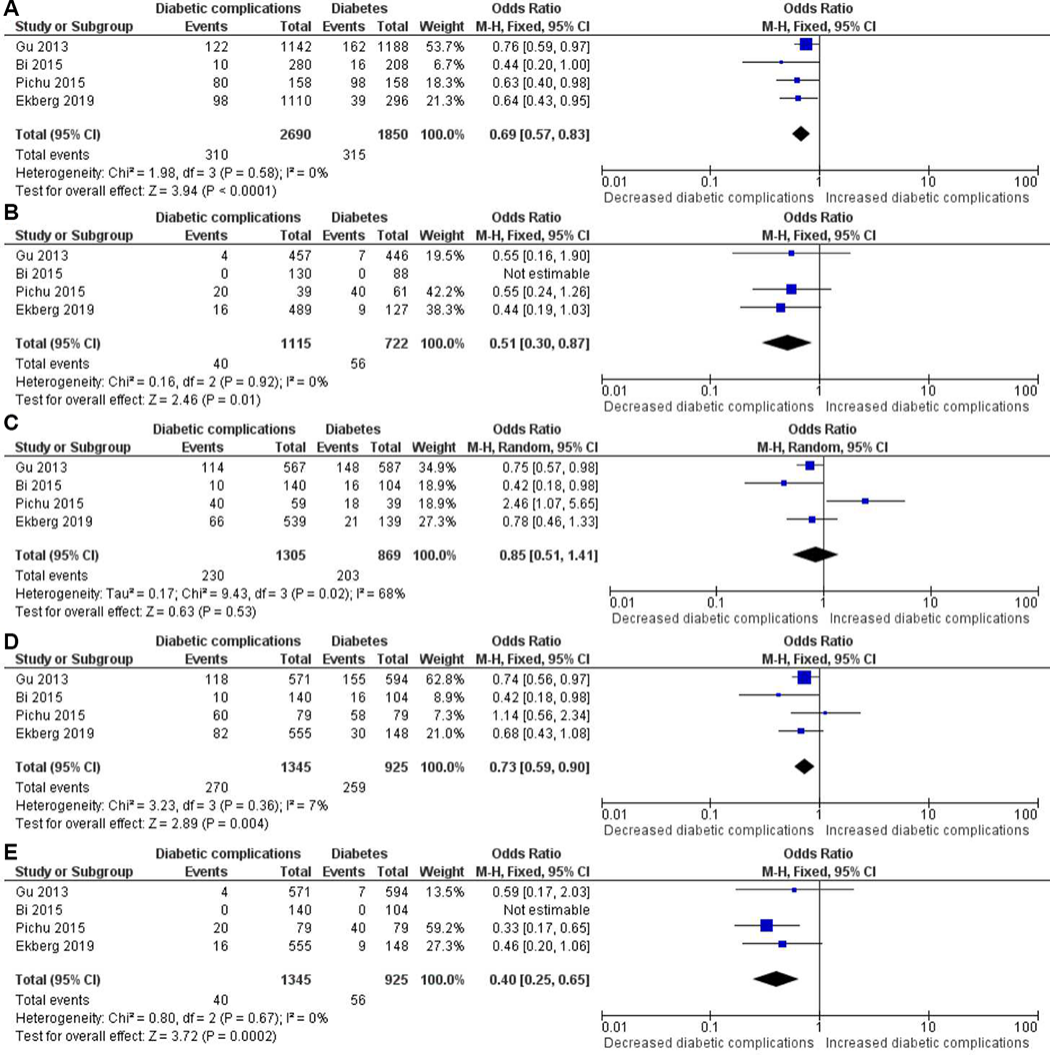
****Forest plot of the meta-analysis for association between the *HIF1A* Pro582Ser genetic polymorphism and risk of diabetic complications under the allelic (**A**), homozygous (**B**), heterozygous (**C**), dominant (**D**) and recessive (**E**) genetic model.

The results of meta-analysis and heterogeneity test between *HIF1A* Pro582Ser polymorphism and the risk of diabetic complications were summarized in detail in Table 2 and Figure 5. The pooled analysis indicated that the *HIF1A* Pro582Ser polymorphism was also significantly associated with a decreased risk of diabetic complications under the allelic (OR = 0.69, 95% CI = 0.57-0.83; *P* < 0.001), homozygous (OR = 0.51, 95% CI = 0.30-0.87; *P* = 0.014), recessive (OR = 0.73, 95% CI = 0.59-0.90; *P* = 0.004) and dominant (OR = 0.40, 95% CI = 0.25-0.65; *P* < 0.001) genetic models. Both the Cochran’s Q test and estimate of I^2^ showed no significant heterogeneity among the constituent studies under the allelic (*P*_heterogeneity_ = 0.577, I^2^ = 0%), homozygous (*P*_heterogeneity_ = 0.923, I^2^ = 0%), recessive (*P*_heterogeneity_ = 0.358, I^2^ = 7%) and dominant (*P*_heterogeneity_ = 0.671, I^2^ = 0%) genetic models. In contrast, no significant association was found under the heterozygous genetic model (OR = 0.85, 95% CI = 0.51-1.41; *P* = 0.532). However, significant between-study heterogeneity was found under the heterozygous genetic model (*P*_heterogeneity_ = 0.024, I^2^ = 68.2%).

**Figure 6:**
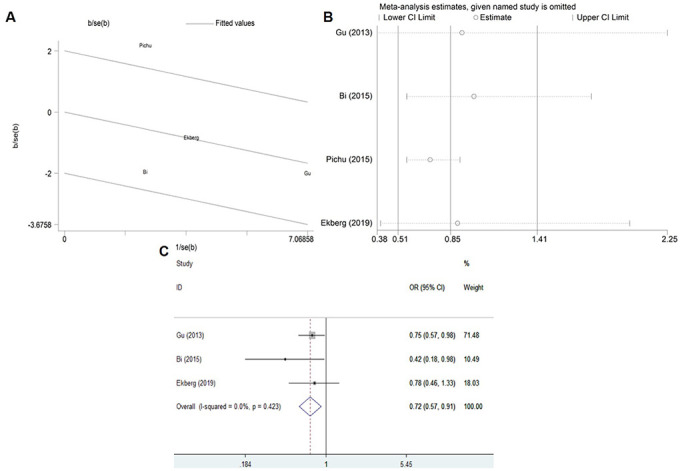
Galbraith plot (**A**), Sensitivity analysis (**B**) and Corrected ORs (**C**) for the association between the *HIF1A* Pro582Ser genetic polymorphism and risk of diabetic complications under the heterozygous genetic model.

Similarly, Galbraith plot analysis and sensitivity analysis were used to detect the possible sources of heterogeneity (Figure 6). Galbraith plot analysis revealed that the Pichu et al. study was the outlier (Figure 6A), which was consistent with the results of sensitivity analysis (Figure 6B). Interestingly, the significant heterogeneity was eliminated after omitting the study by Pichu et al. in the meta-analysis under the heterozygous genetic model (*P*_heterogeneity_ = 0.423, I^2^ = 0%) (Table 3 and Figure 6C).

What’s more, the corrected OR also revealed a significant association between *HIF1A* Pro582Ser polymorphism and a decreased risk of diabetic complications under the heterozygous genetic model (OR = 0.72, 95% CI = 0.57-0.91; *P* = 0.006) (Table 3 and Figure 6C).

### Quantitative synthesis of the association between *HIF1A* Ala588Thr genetic polymorphism with risk of diabetes

**Figure 7:**
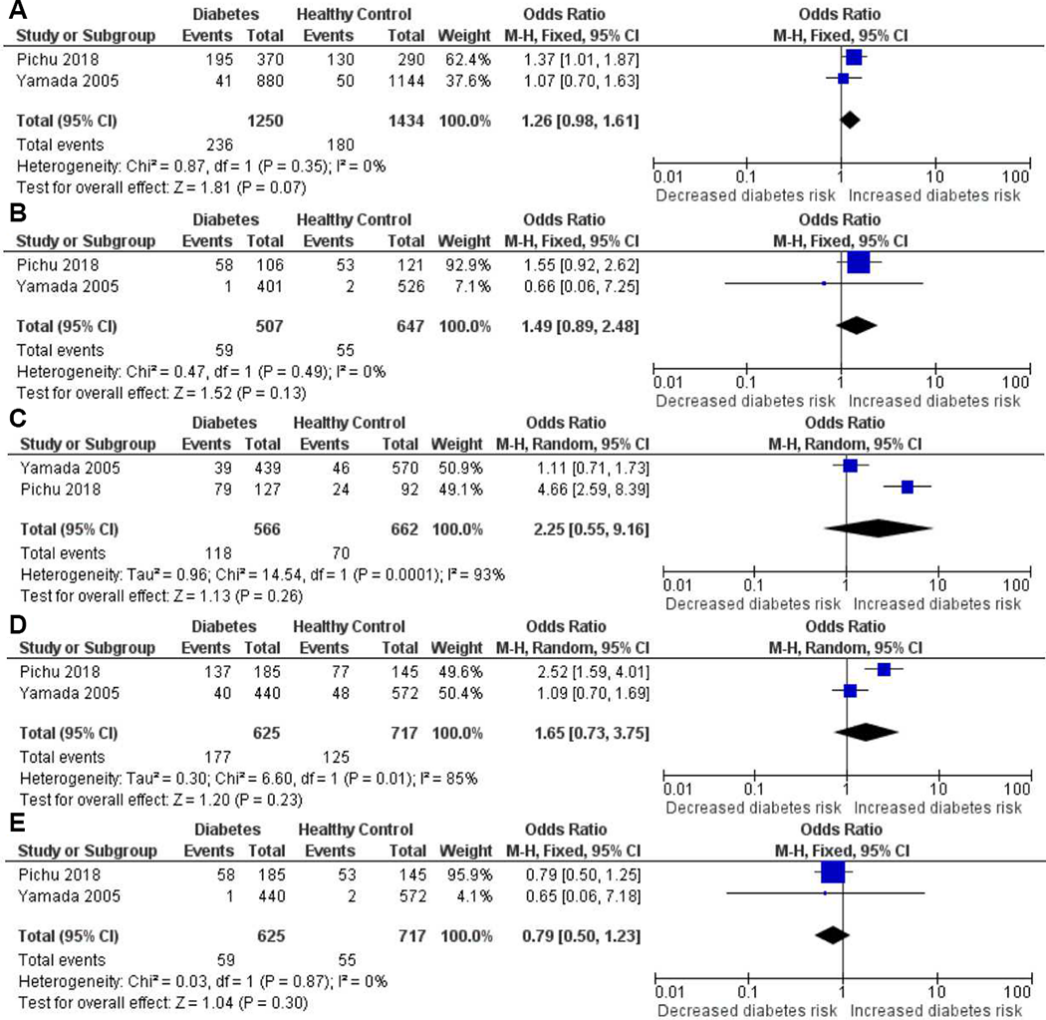
Forest plot of the meta-analysis for association between the *HIF1A* Ala588Thr genetic polymorphism and diabetes risk under the allelic (**A**), homozygous (**B**), heterozygous (**C**), dominant (**D**) and recessive (**E**) genetic model.

The results of meta-analysis and heterogeneity test between *HIF1A* Ala588Thr polymorphism and the risk of diabetes were summarized in detail in Table 4 and Figure 7. The pooled analysis indicated no significant association of *HIF1A* Ala588Thr polymorphism with diabetes risk under all genetic models, including the allelic (OR = 1.26, 95% CI = 0.98-1.61; *P* = 0.07), homozygous (OR = 1.49, 95% CI = 0.89-2.48; *P* = 0.128), heterozygous (OR = 2.25, 95% CI = 0.55-9.16; *P* = 0.259), dominant (OR = 1.65, 95% CI = 0.73-3.75; *P* = 0.229) and recessive (OR = 0.79, 95% CI = 0.50-1.24; *P* = 0.297) genetic models.

**Table 4:** Summary of meta-analysis of association of the *HIF1A* Ala588Thr (rs11549465) genetic polymorphism with risk of diabetes and diabetic complications.

**Genetic model**	**Pooled analysis**					**Tests of heterogeneity**	
	**Pooled OR(95%CI)**	**Z-value**	***P*-value**	**N**	**Model**	***P*-value**	**I^2^%**
Diabetes risk							
Allelic genetic model	1.26 (0.98-1.61)	1.81	0.07	2	F	0.351	0.00%
Homozygous genetic model	1.49 (0.89-2.48)	1.52	0.128	2	F	0.492	0.00%
Heterozygous genetic model	2.25 (0.55-9.16)	1.13	0.259	2	R	<0.001	93.10%
Dominant genetic model	1.65 (0.73-3.75)	1.2	0.229	2	R	0.01	84.90%
Recessive genetic model	0.79 (0.50-1.24)	1.04	0.297	2	F	0.873	0.00%
Diabetic complications risk							
Allelic genetic model	0.85 (0.38-1.92)	0.38	0.701	2	R	<0.001	91.90%
Homozygous genetic model	0.73 (0.15-3.67)	0.38	0.703	2	R	0.012	84.30%
Heterozygous genetic model	0.82 (0.39-1.72)	0.52	0.601	2	R	0.028	79.20%
Dominant genetic model	0.84 (0.34-2.10)	0.37	0.712	2	R	0.004	87.90%
Recessive genetic model	0.77 (0.21-2.85)	0.39	0.696	2	R	0.032	78.30%

Abbreviations: CI: confidence interval; F: fixed-effects model; N: the number of the studies in the meta-analysis; OR: odds ratio; R: random-effects model; Allelic genetic model: A vs. G; Homozygous genetic model: AA vs. GG; Heterozygous genetic model: GA vs. GG; Dominant genetic model: AA + GA vs. GG; Recessive genetic model: AA vs. GA + GG. Significant *P*-value in the pooled analysis are in bold.

### Quantitative synthesis of the association between *HIF1A* Ala588Thr genetic polymorphism with risk of diabetic complications

**Figure 8:**
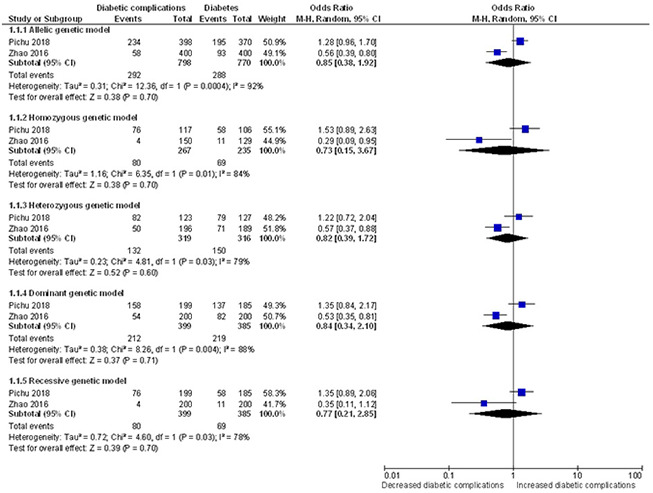
****Forest plot of the meta-analysis for association between the *HIF1A* Ala588Thr genetic polymorphism and risk of diabetic complications under the allelic (**A**), homozygous (**B**), heterozygous (**C**), dominant (**D**) and recessive (**E**) genetic model.

The results of meta-analysis and heterogeneity test between *HIF1A* Ala588Thr polymorphism and the risk of diabetic complications were summarized in detail in Table 4 and Figure 8. No effects of the *HIF1A* Pro582Ser polymorphism were also found in risk of diabetic complications under the allelic (OR = 0.85, 95% CI = 0.38-1.92; *P* = 0.701), homozygous (OR = 0.73, 95% CI = 0.15-3.67; *P* = 0.703), heterozygous (OR = 0.82, 95% CI = 0.39-1.72; *P* = 0.601), dominant (OR = 0.84, 95% CI = 0.34-2.10; *P* = 0.712) and recessive (OR = 0.77, 95% CI = 0.21-2.85; *P* = 0.696) genetic models.

### Publication bias evaluation

**Figure 9:**
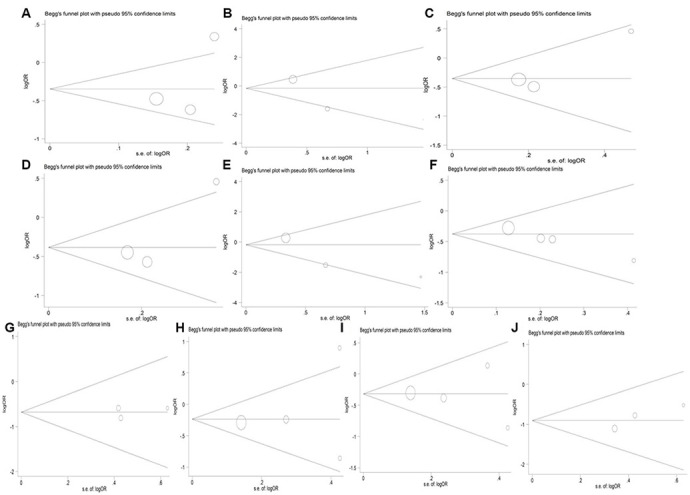
Begg’s funnel plot for studies of the *HIF1A* Pro582Ser genetic polymorphism in diabetes risk under the allelic (**A**), homozygous (**B**), heterozygous (**C**), dominant (**D**) and recessive (**E**) genetic model, and in risk of diabetic complications under the allelic (**F**), homozygous (**G**), heterozygous (**H**), dominant (**I**) and recessive (**J**) genetic model.

Publication bias of the included studies was assessed by using the Begg’s funnel plot (Figure 9). For the meta-analysis of the association between *HIF1A* Pro582Ser polymorphism and the risk of diabetes, no evidence of significant publication bias was detected by the Begg’s test (*P* = 0.089 for allelic genetic model; *P* = 0.602 for homozygous genetic model; *P* = 0.734 for dominant genetic model; *P* = 0.296 for recessive genetic model). The P-values for Begg’s test also demonstrated that there was no publication bias of meta-analysis of the association between *HIF1A* Pro582Ser polymorphism and the risk of diabetic complications (*P* > 0.1 for all genetic models).

## DISCUSSION

To our knowledge, this is the first meta-analysis to explore the genetic associations between *HIF1A* polymorphisms (Pro582Ser and Ala588Thr) and the occurrence of diabetes and diabetic complications. The results indicated *HIF1A* Pro582Ser polymorphism was significantly associated with reduced risk of diabetes under the heterozygous genetic model. Furthermore, after excluding the outlier study that deviated from Hardy-Weinberg equilibrium (HWE) in controls and contributed to between-study heterogeneity, the corrected ORs demonstrated that *HIF1A* Pro582Ser polymorphism could perform a protective effect on the risk of diabetes under all other genetic models. For the meta-analysis of diabetic complications, the findings provided evidence for the protective associations between *HIF1A* Pro582Ser polymorphism and diabetic complications under all genetic models except the heterozygous genetic model. Similarly, when the outlier study was removed, the corrected ORs also showed a protective association under the heterozygous genetic model. However, no significant effects of the *HIF1A* Ala588Thr polymorphism were found in the risk of diabetes and diabetic complications.

As a key oxygen sensor mediating cellular adaptive responses to hypoxia, HIF-1α plays a pivotal role in cellular and systemic homeostatic. The stabilization of HIF-1α is regulated by oxygen-dependent prolyl hydroxylation of proline domains located in Pro402 and Pro564, which is significant for the effect of hyperglycemia on HIF-1α [8, 34]. However, HIF-1α Pro582 has not been certified as a domain for hydroxylating, and the substitution of serine for proline in this position has no essential role in HIF-1α stability [27, 29, 35]. Amino acids 582 is contained in a region of HIF-1α subunit which could act independently to convey inducible responses and confer transcriptional activation [36, 37]. Previous studies revealed that *HIF1A* Pro582Ser polymorphism was a stable variant and showed increased transcriptional activity, which may offer enhanced HIF-1α activity under normoxic conditions [38, 39]. It has been postulated that the enhanced activity of HIF-1α may provide increased adaptability for pseudohypoxia induced by hyperglycemia [12, 19, 40]. Hence, the increased transcriptional activity of HIF-1α but not stability may provide a functional explanation for the protective effect of *HIF1A* Pro582Ser mutation on the risk of diabetes and diabetic complications.

Heterogeneity among constituent studies is common in the meta-analysis of genetic association study and may affect the interpretation of the meta-analysis results [41, 42]. For the meta-analysis of the role of *HIF1A* Pro582Ser polymorphism in the risk of diabetic complications, one of the strengths was lack of obvious heterogeneity in all genetic models except the heterozygous genetic model. In contrast, for the meta-analysis of the *HIF1A* Pro582Ser polymorphism in the risk of diabetes, significant heterogeneity was found in all genetic models except the heterozygous genetic model. Heterogeneity may result from the potential differences across the included studies, such as the definition of disease, ethnicity, genotyping methods, sample size. To detect the potential sources of heterogeneity, Galbraith plot analysis was firstly used to explore whether there was outlier study. Then, sensitivity analysis by omitting one individual study each time was further performed to identify the possible source of heterogeneity [42]. Galbraith plot analysis indicated the study conducted by Pichu et al. was the outlier, and sensitivity analysis also found Pichu’s study was the main contributor to the significant heterogeneity. We found that *HIF1A* Pro582Ser genotype frequencies showed significant departure of HWE in the health control group of Pichu’s study (*P* = 9.34*10^-7^). In population study, migration, selection, mutation, and absence of random mating may exist when the genotype distribution of controls (disease-free subjects) deviates from HWE. Consequently, the Pichu’s study departure from HWE may bias the meta-analysis results, and can explain the between-study heterogeneity. After excluding the outlier study, the between-study heterogeneity can be effectively eliminated. What's more, the meta-analysis based on the corrected ORs also revealed that the *HIF1A* Pro582Ser polymorphism played a protective role in the risk of diabetes and diabetic complications.

Some limitations of the current meta-analysis should be admitted. Firstly, the development of diabetes and diabetic complications is affected not only by environmental factors but also multiple genetic factors, the effect of gene-to-gene interactions should be taken into account. For example, vascular endothelial growth factor, another susceptibility gene for diabetes, may interact with *HIF1A* gene [22]. Then, because these information was not available in the included studies, the results of our meta-analysis were all based on the crude ORs with corresponding 95% CIs. In addition, due to the small number of studies included in the meta-analysis of *HIF1A* Ala588Thr polymorphism with the risk of diabetes and its complications, the findings should be interpreted with caution. The small sample size may be responsible for the negative relationship between the *HIF1A* Ala588Thr polymorphism and diabetes and diabetic complications.

In conclusion, our meta-analysis revealed the protective role of the *HIF1A* Pro582Ser polymorphism against diabetes and diabetic complications. However, there was no significant association of *HIF1A* Ala588Thr polymorphism with the risk of diabetes and its complications. Owing to the limitations mentioned above, further studies, with larger sample sizes on the association of *HIF1A* genetic polymorphisms (especially *HIF1A* Ala588Thr) with the risk of diabetes and its complications, should be performed to confirm our findings in the future.

## MATERIALS AND METHODS

### Search strategy and inclusion criteria

A systematical literature search was conducted in the following electronic databases: PubMed, Embase, WanFang Data, and China National Knowledge Infrastructure (CNKI) from their starting dates to December 2019. The following keywords used for the search strategy were hypoxia-inducible factor-1α gene (*HIF1A*) or variations (e.g.,“polymorphism”, “single nucleotide polymorphism”, “SNP”, “variant”, “mutation”, “variation”) in combination with diabetes and diabetic complications (e.g., “diabetes mellitus”, “diabetic complications”). Additionally, other possible original publications were identified by manually searching the reference lists of the selected reviews and articles.

All the identified studies were independently evaluated by two investigators according to the inclusion criteria. The included studies met the criteria as follows: (1) studies were conducted in humans and assessed with a case-control design. (2) the association between Pro582Ser and Ala588Thr of *HIF1A* gene and risk of diabetes and diabetic complications was explored. (3) published in English or Chinese. (4) detailed *HIF1A* genotyping data were offered in case and control groups. If the two reviewers disagreed about the inclusion of a study, it was resolved by group discussion or consensus with a third reviewer.

### Data extraction

For the included articles in this study, data were collected by two reviewers independently. The following information was extracted from each publication: last name of the first author, year of publication, country of the study, ethnicity of the population, mean age, gender distribution of cases and controls, allele and genetic distributions in case and control groups, total number of cases and controls.

### Statistical analysis

In this meta-analysis, five genetic models were performed including the allelic (T vs. C of *HIF1A* Pro582Ser gene polymorphism; A vs. G of *HIF1A* Ala588Thr gene polymorphism), homozygous (TT vs. CC of *HIF1A* Pro582Ser gene polymorphism; AA vs. GG of *HIF1A* Ala588Thr gene polymorphism), heterozygous (CT vs. CC of *HIF1A* Pro582Ser gene polymorphism; GA vs. GG of *HIF1A* Ala588Thr gene polymorphism), dominant (TT + CT vs. CC of *HIF1A* Pro582Ser gene polymorphism; AA + GA vs. GG of *HIF1A* Ala588Thr gene polymorphism) and recessive (TT vs. CT + CC of *HIF1A* Pro582Ser gene polymorphism; AA vs. GG + GA of *HIF1A* Ala588Thr gene polymorphism).

The strength of the association between *HIF1A* gene polymorphisms and diabetes and diabetic complications risk was evaluated by odds ratios (ORs) with 95% confidence intervals (CIs) according to the alleles and genotypes in case and control groups. The pooled estimates of the OR were calculated from a combination of studies in the allelic, homozygous, heterozygous, recessive and dominant models, respectively. The Z test was applied to determine the statistical significance of the pooled OR. The I^2^ metric and Cochran’s Q test were conducted to check the possibility of heterogeneity among the included studies. Between-study heterogeneity was considered as a statistic significance at I^2^ > 50% for the I^2^ test and *P* < 0.05 for the Q statistics [43]. If significant heterogeneity existed, the pooled OR was calculated via random effect model (the DerSimonian and Laird method). Otherwise, the fixed effect model (the Mante-Haenszel method) was used. Sensitivity analysis and Galbraith plot were conducted to explore the potential sources of heterogeneity across the studies. Potential publication bias was assessed with Begg’s test [44]. All statistical analyses in our study were conducted using the software Review Manager 5.0 and STATA version 12.0 (Stata Corp, College Station, TX, USA).
